# Supplementary material for: Agonistic Bivalent Human scFvs-Fcγ Fusion Antibodies to OX40 Ectodomain Enhance T Cell Activities against Cancer
Source: Vaccines (Basel). 2023 Dec 7;11(12):1826. doi: 10.3390/vaccines11121826 (PMC10747724; doi:10.3390/vaccines11121826)
Supplement: Supplementary file 1 [file vaccines-11-01826-s001.zip › vaccines-2723124-supplementary.pdf]

Supplement information

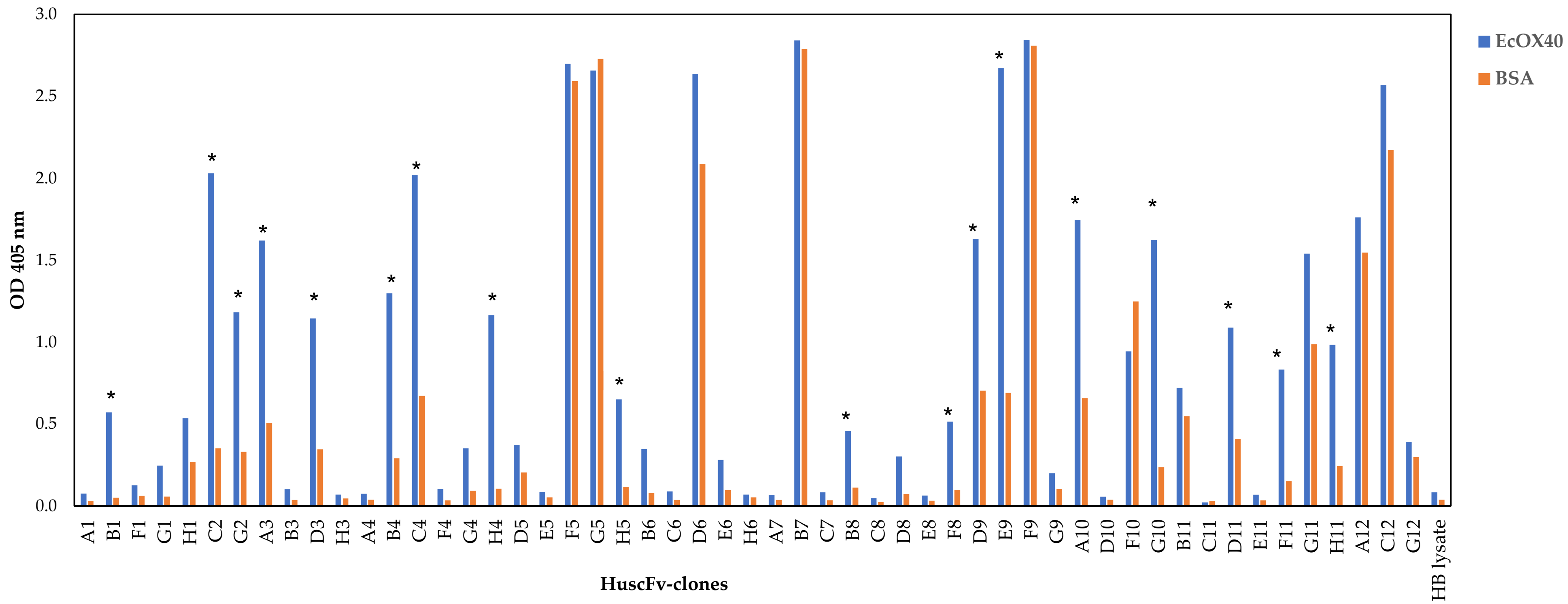

Figure S1 Indirect ELISA results for detecting the binding of HuscFvs in lysates of 53 *huscfv*-phagemid transformed HB2151 *E. coli* clones derived from the phage panning with functionally active recombinant EcOX40 protein. Lysates of 18 *E. coli* clones: B1, C2, G2, A3, D3, B4, C4, H4, H5, B8, F8, D9, E9, A10, G10, D11, F11 and H11 (asterisks) gave OD 405 nm to the EcOX40 two times higher than to BSA control. HB lysate, lysate of normal HB2151 *E. coli* served as negative control.

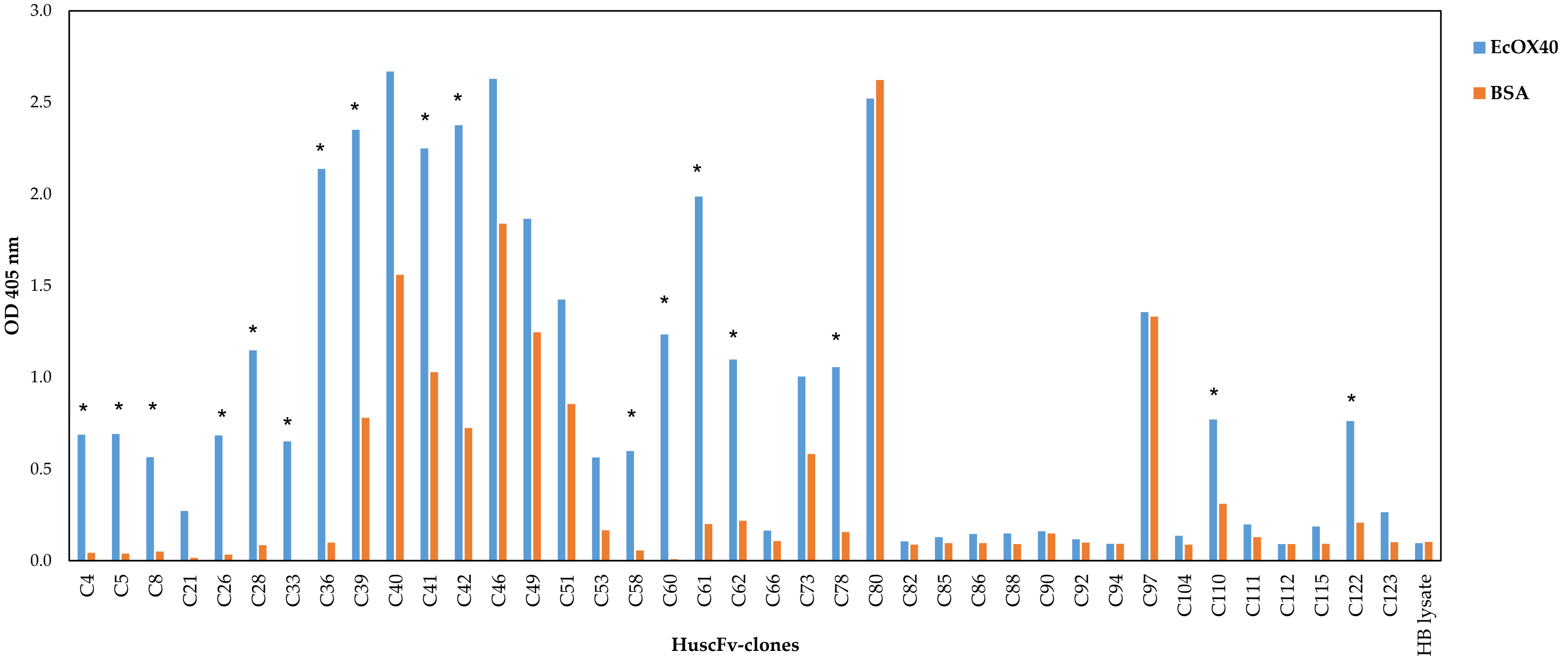

Figure S2 Indirect ELISA results for detecting the binding of HuscFvs in lysates of 39 *huscfv*-phagemid transformed HB2151 *E. coli* clones derived from phage panning with HEK-OX40 cells. Lysates of 17 *E. coli* clones (no. C4, C5, C8, C26, C28, C33, C36, C39, C41, C42, C58, C60, C61, C62, C78, C110 and C122) gave OD 405 nm to recombinant EcOX40 two times higher than to BSA control (asterisks). HB lysate, lysate of normal HB2151 *E. coli* served as negative control.

Table S1 Percent amino acid homology of the HuscFv sequences from the *huscfv*-phagemid-transformed-HB2151 *E. coli* clones C2, D11, F8, C36, C41, C62 and C78 with the closest human V region frameworks (FRs).

HuscFv clone	VDJ gene and allele identification		Score	Identity
C2-VH	V-gene and allele	Homsap IGHV1-2*02 F	1426	99.65% (287/288 nt)
	J-gene and allele	Homsap IGHJ6*02 F	247	88.71% (55/62 nt)
	D-gene and allele by IMGT/junction analysis	Homsap IGHD2-2*03 F	D-region is in reading frame 3	
	FR-IMGT lengths, CDR-IMGT lengths and AA junction	[25.17.38.11]	[8.8.19]	CARDMDIVVVPAAYYYGMDVW
C2-VL	V-gene and allele	Homsap IGKV1-9*01 F	1174	91.40% (255/279 nt)
	J-gene and allele	Homsap IGKJ1*01 F	158	91.89% (34/37 nt)
	FR-IMGT lengths, CDR-IMGT lengths and AA junction	[26.17.36.10]	[6.3.9]	CHQTSRLPWTF
D11-VH	V-gene and allele	Homsap IGHV1-18*04 F	1300	94.79% (273/288 nt)
	J-gene and allele	Homsap IGHJ6*02 F	247	88.71% (55/62 nt)
	D-gene and allele by IMGT/junction analysis	Homsap IGHD2-2*01 F	D-region is in reading frame 2	
	FR-IMGT lengths, CDR-IMGT lengths and AA junction	[25.17.38.11]	[8.8.19]	CARWYCSSSSCTTYYYGMDVW
D11-VL	V-gene and allele	Homsap IGKV1-39*01 F, or Homsap IGKV1D-39*01 F	1354	98.57% (275/279 nt)
	J-gene and allele	Homsap IGKJ1*01 F	131	83.78% (31/37 nt)
	FR-IMGT lengths, CDR-IMGT lengths and AA junction	[26.17.36.10]	[6.3.10]	CQQSYSTPPGTF
F8-VH	V-gene and allele	Homsap IGHV5-51*01 F	1201	90.97% (262/288 nt)
	J-gene and allele	Homsap IGHJ4*02 F	172	85.11% (40/47 nt)
	D-gene and allele by IMGT/junction analysis	Homsap IGHD1-7*01 F	D-region is in reading frame 2	
	FR-IMGT lengths, CDR-IMGT lengths and AA junction	[25.17.38.11]	[8.8.11]	CAILDLRVGFDYR (TRP 118 not identified)
F8-VL	V-gene and allele	Homsap IGKV3-20*01 F	1216	92.20% (260/282 nt)
	J-gene and allele	Homsap IGKJ2*02 F	149	89.19% (33/37 nt)
	FR-IMGT lengths, CDR-IMGT lengths and AA junction	[26.17.36.10]	[7.3.9]	CQQYYTYPWTF

HuscFv clone	VDJ gene and allele identification		Score	Identity
C36-VH	V-gene and allele	Homsap IGHV3-9*01 F	1327	95.83% (276/288 nt)
	J-gene and allele	Homsap IGHJ6*02 F	220	83.87% (52/62 nt)
	D-gene and allele by IMGT/junction analysis	Homsap IGHD3-10*01 F	D-region is in reading frame 2	
	FR-IMGT lengths, CDR-IMGT lengths and AA junction	[25.17.38.11]	[8.8.16]	CAKDSGPYGSGHYGMDVW
C36-VL	V-gene and allele	Homsap IGKV6-21*01 F, or Homsap IGKV6D-21*01 F	1093	88.17% (246/279
	J-gene and allele	Homsap IGKJ1*01 F	185	91.89% (34/37 nt)
	FR-IMGT lengths, CDR-IMGT lengths and AA junction	[26.17.36.10]	[6.3.9]	CHQTSASPWTF
C41-VH	V-gene and allele	Homsap IGHV1-18*01 F	1399	98.61% (284/288 nt)
	J-gene and allele	Homsap IGHJ6*02 F	247	88.71% (55/62 nt)
	D-gene and allele by IMGT/junction analysis	Homsap IGHD2-2*03 F	D-region is in reading frame 3	
	FR-IMGT lengths, CDR-IMGT lengths and AA junction	[25.17.38.11]	[8.8.21]	CARDLGPVVVPAAILDYYGMDVW
C41-VL	V-gene and allele	Homsap IGKV1-39*01 F, or Homsap IGKV1D-39*01 F	1363	98.92% (276/279 nt)
	J-gene and allele	Homsap IGKJ1*01 F	167	94.59% (35/37 nt)
	FR-IMGT lengths, CDR-IMGT lengths and AA junction	[26.17.36.10]	[6.3.8]	CQQSYSTWTF
C62-VH	V-gene and allele	Homsap IGHV3-30-3*01 F	1318	95.49% (275/288 nt)
	J-gene and allele	Homsap IGHJ4*02 F	190	89.36% (42/47 nt)
	D-gene and allele by IMGT/junction analysis	Homsap IGHD2-15*01 F	D-region is in reading frame 2	
	FR-IMGT lengths, CDR-IMGT lengths and AA junction	[25.17.38.11]	[8.8.18]	CVRDTAPCSGGSCHRDLDYW
C62-VL	V-gene and allele	Homsap IGKV1-27*01 F	1354	98.57% (275/279 nt)
	J-gene and allele	Homsap IGKJ2*01 F	176	97.30% (36/37 nt)
	FR-IMGT lengths, CDR-IMGT lengths and AA junction	[26.17.36.10]	[6.3.9]	CQKYNSAPYTF

HuscFv clone	VDJ gene and allele identification		Score	Identity
C78-VH	V-gene and allele	Homsap IGHV5-10-1*03 F	1264	93.40% (269/288 nt)
	J-gene and allele	Homsap IGHJ6*02 F	256	90.32% (56/62 nt)
	D-gene and allele by IMGT/junction analysis	Homsap IGHD5-18*01 F	D-region is in reading frame 3	
	FR-IMGT lengths, CDR-IMGT lengths and AA junction	[25.17.38.11]	[8.8.18]	CARREGGYTFGFHYYGMDVW
C78-VL	V-gene and allele	Homsap IGKV3-15*01 F	1381	99.64% (278/279 nt)
	J-gene and allele	Homsap IGKJ3*01 F	166	97.14% (34/35 nt)
	FR-IMGT lengths, CDR-IMGT lengths and AA junction	[26.17.36.10]	[6.3.9]	CQQYNNWPLTF

Ig, immunoglobulin; FR, immunoglobulin framework region; VH, variable domain of heavy chain; VL, variable domain of light chain. An asterisk followed by two numbers indicates the allele polymorphism.

Table S2 Ramachandran plot analysis of the HuscFv models.

Plot statistics	EcOX40		HuscFvC2		HuscFvD11		HuscFvF8		HuscFvC36		HuscFvC41		HuscFvC61		HuscFvC78	
Residues in most favored regions [A, B, L]	207	90.4%	183	90.1%	180	91.8%	180	91.8%	183	92.9%	187	90.8%	184	90.6%	181	90.5%
Residues in additional allowed regions [a,b,l,p]	20	8.7%	19	9.4%	13	6.6%	13	6.6%	11	5.6%	14	6.8%	14	6.9%	17	8.5%
Residues in generously allowed regions [~a,~b,~l,~p]	2	0.9%	0	0%	2	1.0%	2	1.0%	2	1%	1	0.5%	2	1.0%	2	1.0%
Residues in disallowed regions	0	0%	1	0.5%	1	0.5%	1	0.5%	1	0.5%	4	1.9%	3	1.5%	0	0%
Number of non-glycine and non-proline residues	229	100%	203	100%	196	100%	196	100%	197	100%	206	100%	203	100%	200	100%
Number of end-residues (excl. Gly and Pro)	4		2		2		2		2		2		2		2	
Number of glycine residues (shown as triangles)	17		34		32		32		33		32		34		34	
Number of proline residues	16		9		11		11		13		9		8		11	
Total number of residues	266		248		241		241		245		249		247		247	

Table S3. Ectodomain of OX40 (EcOX40) residues and site(s) that interacted with the HuscFvC2, D11, F8, C36, C41, C62 and C78.

EcOX40		HuscFvC2		Interaction Bond
Domain	Residue	Domain	Residue	
CRD2	CYS53	VH-CDR2	SER55	Hydrogen
CRD2	PRO41	VH-CDR3	TYR110	Hydrogen
CRD2	LEU61	VL-CDR3	ARG234	Hydrogen
CRD2	TRP58	VL-CDR3	LEU235	Hydrogen
CRD1	ARG37	VH-CDR2	ASN54	Hydrogen
CRD1	ARG37	VH-CDR2	ASN54	Hydrogen
CRD2	ARG67	VL-CDR3	SER233	Hydrogen
CRD2	ARG67	VL-CDR1	TYR173	Hydrogen
CRD2	ARG67	VL-CDR1	TYR173	Hydrogen
CRD2	ARG67	VL-CDR3	SER233	Hydrogen
CRD2	ASN60	VL-CDR3	ARG234	Hydrogen
CRD2	ARG62	VL-CDR1	GLN168	Hydrogen
CRD2	TRP58	VH-CDR3	ALA107	Hydrophobic
CRD2	PHE43	VH-CDR1	TYR33	Hydrophobic
CRD2	PRO41	VH-CDR3	PRO106	Hydrophobic
CRD2	LEU70	VH-CDR3	ALA107	Hydrophobic

EcOX40		HuscFvD11		Interaction Bond
Domain	Residue	Domain	Residue	
CRD2	CYS56	VH-CDR1	THR27	Hydrogen
CRD2	TRP58	VH-CDR1	THR28	Hydrogen
CRD2	ASN60	VH-CDR1	THR30	Hydrogen
CRD2	PRO41	VH-CDR3	TYR100	Hydrogen
CRD2	GLY42	VH-CDR3	CYS101	Hydrogen
CRD2	TRP58	VH-CDR3	CYS101	Hydrogen
CRD2	ARG67	VH-CDR1	THR30	Hydrogen

EcOX40		HuscFvD11		Interaction Bond
**Domain**	**Residue**	**Domain**	**Residue**	
CRD2	CYS71	VH-CDR3	SER102	Hydrogen
CRD2	ALA73	VH-CDR3	SER103	Hydrogen
CRD3	THR91	VH-CDR1	TYR27	Hydrogen
CRD1	GLY40	VH-CDR3	SER101	Hydrogen
CRD2	ARG67	VH-CDR2	TYR54	Electrostatic
CRD2	LEU61	VH-CDR2	TYR54	Hydrophobic
CRD2	TRP58	VH-CDR3	CYS101	Other
CRD2	TRP58	VH-CDR3	CYS101	Other
CRD3	TYR91	VH-CDR1	TYR27	Hydrophobic
CRD3	PHE41	VH-CDR3	TYR100	Hydrophobic
CRD2	CYS56	VH-CDR3	CYS101	Hydrophobic
CRD2	LEU70	VH-CDR3	CYS101	Hydrophobic
CRD2	PRO41	VL-CDR2	ALA191	Hydrophobic
**EcOX40**		**HuscFvF8**		**Interaction Bond**
**Domain**	**Residue**	**Domain**	**Residue**	
CRD2	TRP58	VL-CDR1	TYR166	Hydrogen
CRD2	PRO41	VL-CDR3	TYR228	Hydrogen
CRD2	TRP58	VL-CDR3	TYR226	Hydrogen
CRD2	ARG67	VL-CDR1	TYR166	Hydrogen
CRD2	ARG67	VH-CDR3	ARG102	Hydrogen
**EcOX40**		**HuscFvC36**		**Interaction Bond**
**Domain**	**Residue**	**Domain**	**Residue**	
CRD2	ARG67	VL-CDR1	ASP170	Electrostatic
CRD2	ARG67	VL-CDR1	ASP170	Electrostatic
CRD2	CYS56	VH-CDR3	TYR103	Hydrogen
CRD2	TRP58	VH-CDR3	SER105	Hydrogen
CRD2	TRP58	VH-CDR3	GLY106	Hydrogen
CRD2	CYS71	VL-CDR2	TYR188	Hydrogen
CRD2	LEU61	VL-CDR3	SER232	Hydrogen

EcOX40		HuscFvC36		Interaction Bond
**Domain**	**Residue**	**Domain**	**Residue**	
CRD2	GLY42	VL-CDR2	TYR188	Hydrogen
CRD2	TRP58	VH-CDR3	TYR103	Hydrogen
CRD2	ARG62	VH-CDR3	HIS107	Hydrogen
CRD2	ARG62	VH-CDR3	SER105	Hydrogen
CRD2	ARG62	VH-CDR3	HIS107	Hydrogen
CRD2	ARG62	VH-CDR2	SER57	Hydrogen
CRD2	ARG67	VL-CDR3	THR229	Hydrogen
CRD2	ARG67	VL-CDR1	ASP170	Hydrogen
CRD2	ARG67	VL-CDR1	ASP170	Hydrogen
CRD2	ARG67	VH-CDR3	TYR108	Electrostatic
CRD2	TRP58	VH-CDR3	PRO102	Hydrophobic
CRD2	TRP58	VH-CDR3	TYR108	Hydrophobic
CRD2	TRP58	VH-CDR3	TYR108	Hydrophobic
CRD2	TRP58	VL-CDR2	TYR188	Hydrophobic
CRD2	TRP58	VH-CDR3	PRO102	Hydrophobic
**EcOX40**		**HuscFvC41**		**Interaction Bond**
**Domain**	**Residue**	**Domain**	**Residue**	
CRD2	PRO41	VH-CDR3	ARG98	Hydrogen
CRD1	ARG37	VH-CDR1	GLY26	Hydrogen
CRD2	TRP58	VH-CDR3	GLY101	Hydrogen
CRD2	TRP58	VH-CDR3	TYR112	Hydrogen
CRD3	TYR91	VH-CDR3	VAL104	Hydrophobic
CRD2	TRP58	VH-CDR3	TYR112	Hydrophobic
**EcOX40**		**HuscFvC62**		**Interaction Bond**
**Domain**	**Residue**	**Domain**	**Residue**	
CRD3	ASP89	VH-CDR2	LYS58	Hydrogen; Electrostatic
CRD3	ASP89	VH-CDR2	LYS58	Hydrogen; Electrostatic
CRD3	GLN86	VH-CDR2	ASN57	Hydrogen
Stalk	GLN152	VH-CDR3	CYS103	Hydrogen

EcOX40		HuscFvC62		Interaction Bond
Domain	Residue	Domain	Residue	
Stalk	GLN152	VH-CDR3	HIS109	Hydrogen
CRD4	GLN111	VH-CDR3	ARG110	Hydrogen
CRD4	GLN111	VH-CDR3	ARG110	Hydrogen
Stalk	GLU150	VL-CDR1	SER170	Hydrogen
Stalk	GLU150	VL-CDR1	ASN172	Hydrogen
CRD4	ASN110	VH-CDR2	LYS58	Hydrogen
Stalk	GLN149	VL-CDR1	ASN172	Hydrogen
Stalk	GLN149	VL-CDR1	ASN171	Hydrogen
Stalk	THR151	VL-CDR1	ASN172	Hydrogen
CRD4	LYS114	VH-CDR3	CYS108	Hydrophobic
**EcOX40**		**HuscFvC78**		**Interaction Bond**
**Domain**	**Residue**	**Domain**	**Residue**	
CRD2	ARG67	VH-CDR2	ASP52	Hydrogen; Electrostatic
CRD2	ARG67	VH-CDR2	ASP55	Hydrogen; Electrostatic
CRD2	ARG67	VH-CDR2	ASP52	Hydrogen; Electrostatic
CRD2	ARG67	VH-CDR2	ASP52	Electrostatic
CRD3	ASP96	VH-CDR3	GLY106	Hydrogen
CRD2	ARG62	VH-CDR3	GLY102	Hydrogen
CRD2	ARG62	VH-CDR1	ASN31	Hydrogen
CRD2	ARG67	VH-CDR2	ASP55	Hydrogen
CRD2	ARG62	VH-CDR1	ASN31	Hydrogen
CRD2	ARG67	VH-CDR2	ASP55	Hydrogen
CRD2	TRP58	VL-CDR3	TRP234	Hydrogen

CRD, cysteine-rich domain; VH, variable domain of heavy chain; VL, variable domain of light chain; CDR, complementarity-determining region

Table S4 Codon optimization of *huscfvs* for mammalian cell expression system.

	Codon Adaptation Index (CAI)	
vh	**Original**	**Optimized**
C2#	0.8	0.94
D11	0.78	0.96
C36	0.76	0.96
C41	0.77	0.96
vl	**Original**	**Optimized**
C2	0.74	0.97
D11	0.76	0.97
C36	0.77	0.96#
C41	0.74	0.97

vh, gene coding for variable domain of heavy chain; *vl*, gene coding for variable domain of light chain
